# Supplementary material for: RalGTPases contribute to Schwann cell repair after nerve injury via regulation of process formation
Source: J Cell Biol. 2019 Jun 14;218(7):2370–87. doi: 10.1083/jcb.201811002 (PMC6605803; doi:10.1083/jcb.201811002)
Supplement: Supplemental Materials (PDF) [file JCB_201811002_sm.pdf]

JCB

Supplemental material

Galino et al., https://doi.org/10.1083/jcb.201811002

JCB

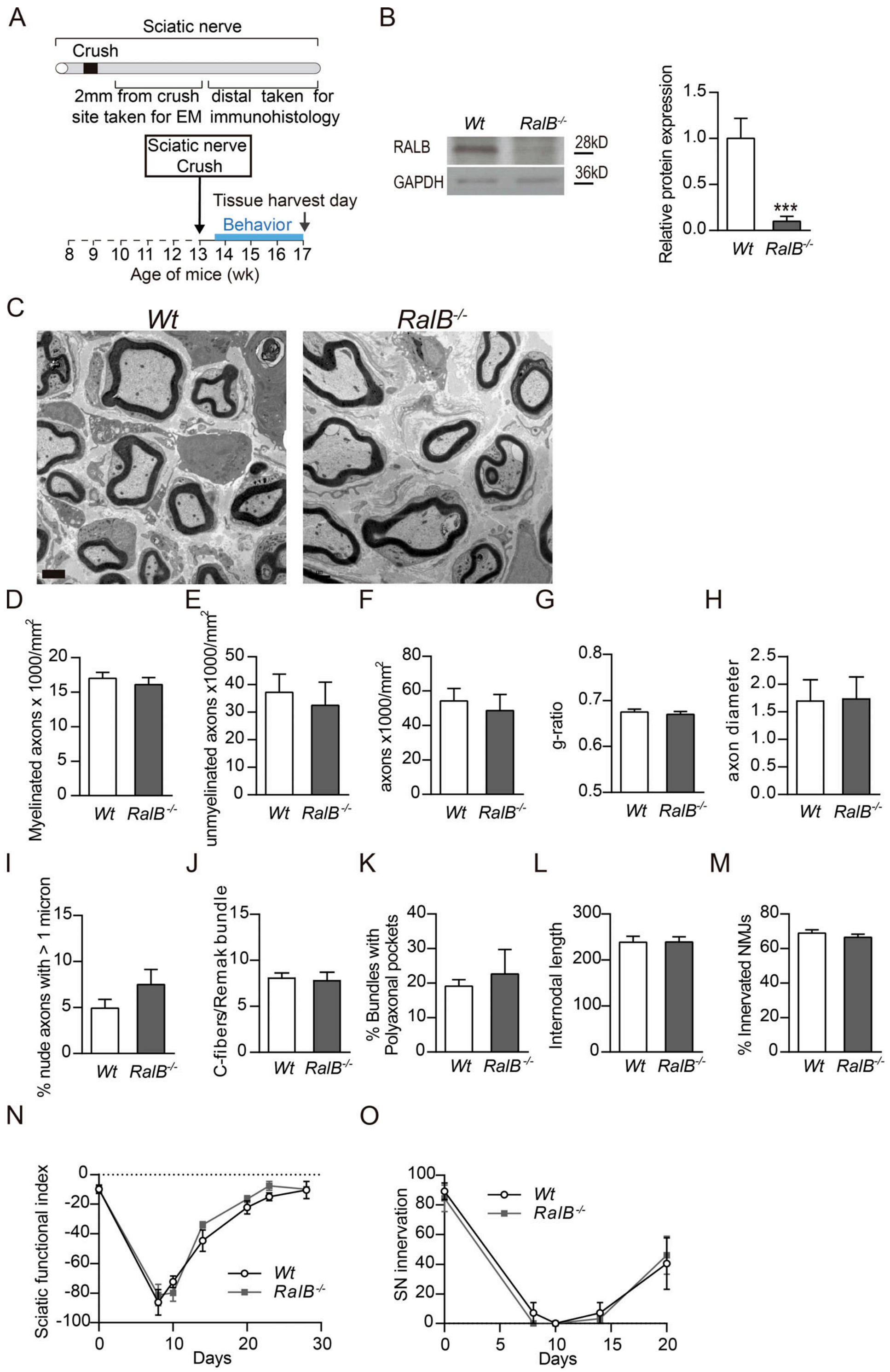

Figure S1. **RalB is dispensable for axon remyelination, reinnervation of distal organs, and functional recovery following peripheral nerve injury. (A)** Schematic representation of the sciatic nerve crush in RalB$^{-/-}$ mice. **(B)** Western blot to analyze the protein expression of RalB in the contralateral sciatic nerve of *RalB*$^{-/-}$ compared with WT mice. **(C)** Electron micrographs of transverse sections of the distal sciatic nerve 1 mo after crush injury in *RalB*$^{-/-}$ and WT mice. Remyelinated axons can be observed, and the degree of remyelination does not differ between genotypes. **(D–F)** Quantification of the density of myelinated (D), nonmyelinated (E), and all axons (F) per square millimeter. **(G)** Quantification of g-ratio 1 mo after injury. **(H)** Quantification of axon diameter 1 mo after injury. **(I)** Quantification of the number of axons with a diameter >1 µm that are nonmyelinated 1 mo after injury. **(J and K)** Study of Remak bundles 1 mo after injury did not show any difference in C-fibers per Remak bundle (J) or the percentage of Remak bundles with polyaxonal pockets (K). **(L)** Quantification of internodal distance. **(M)** Quantification of the number of neuromuscular junctions reinnervated 1 mo after sciatic nerve injury, $n = 4$. **(N)** SFI was used to assess the functional recovery following sciatic nerve crush and did not differ between genotypes. **(O)** Pin prick test was used to analyze the sciatic nerve innervation of the skin following sciatic nerve crush and did not differ between genotypes. For behavioral studies, $n = 8–12$. Data are presented as mean ± SEM (***$P < 0.001$, Student's t test). Scale bar, 2 µm.

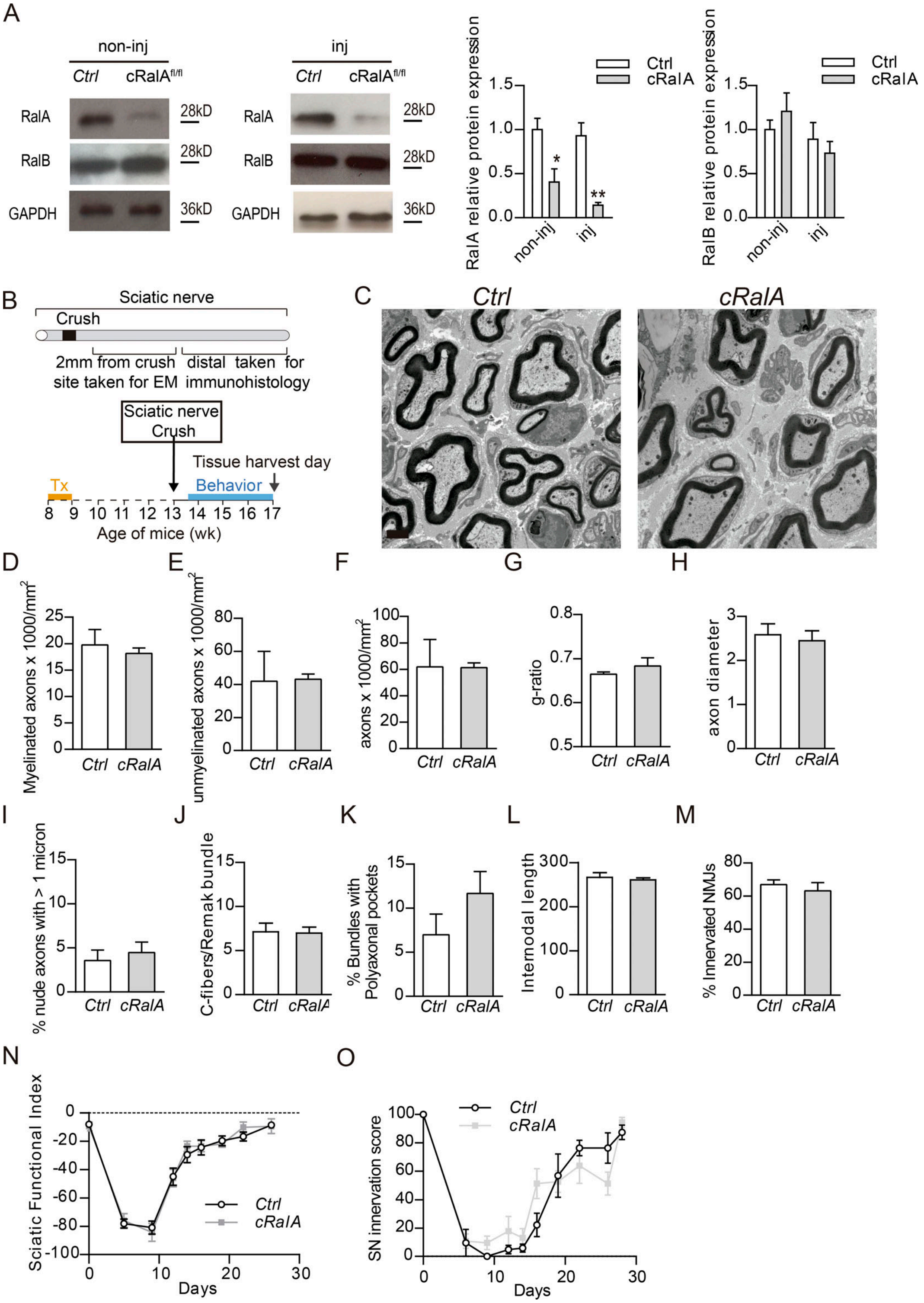

Figure S2. **Conditional KO of RalA (*cRalA*) in SCs does not affect repair after nerve injury. (A)** Western blot to analyze the expression of RalA in noninjured (non-inj) and injured (inj) nerves of *cRalA* versus control mice. RalB expression is also shown. GAPDH was used as loading control. **(B)** Schematic representation of the nerve crush injury in cRalA mice. **(C)** Electron micrographs of transverse sections of the distal sciatic nerve 1 mo after crush injury in ctrl and *cRalA*. **(D–F)** Quantification of the density of myelinated (D), nonmyelinated (E), and all axons (F) per square millimeter. **(G)** Quantification of g-ratio 1 mo after injury. **(H)** Quantification of axon diameter 1 mo after injury. **(I)** Quantification of nonmyelinated axons of >1 µm diameter 1 mo after injury. **(J and K)** Study of Remak bundles 1 mo after injury did not show any difference in C-fibers per Remak bundle (J) or in the percetage of Remak bundles with polyaxonal pockets (K). **(L)** Quantification of inter-nodal distance. **(M)** Quantification of the number of neuromuscular junctions reinnervated 1 mo after sciatic nerve injury. *n* = 4. **(N)** SFI was used to assess the functional recovery following sciatic nerve crush and did not differ between genotypes. **(O)** Pin prick test was used to analyze the sciatic nerve innervation of the skin following sciatic nerve crush and did not differ between genotypes. For behavioral studies, *n* = 8–12. Data are presented as mean ± SEM (*, $P < 0.05$, Student's *t* test). Scale bar, 2 µm.

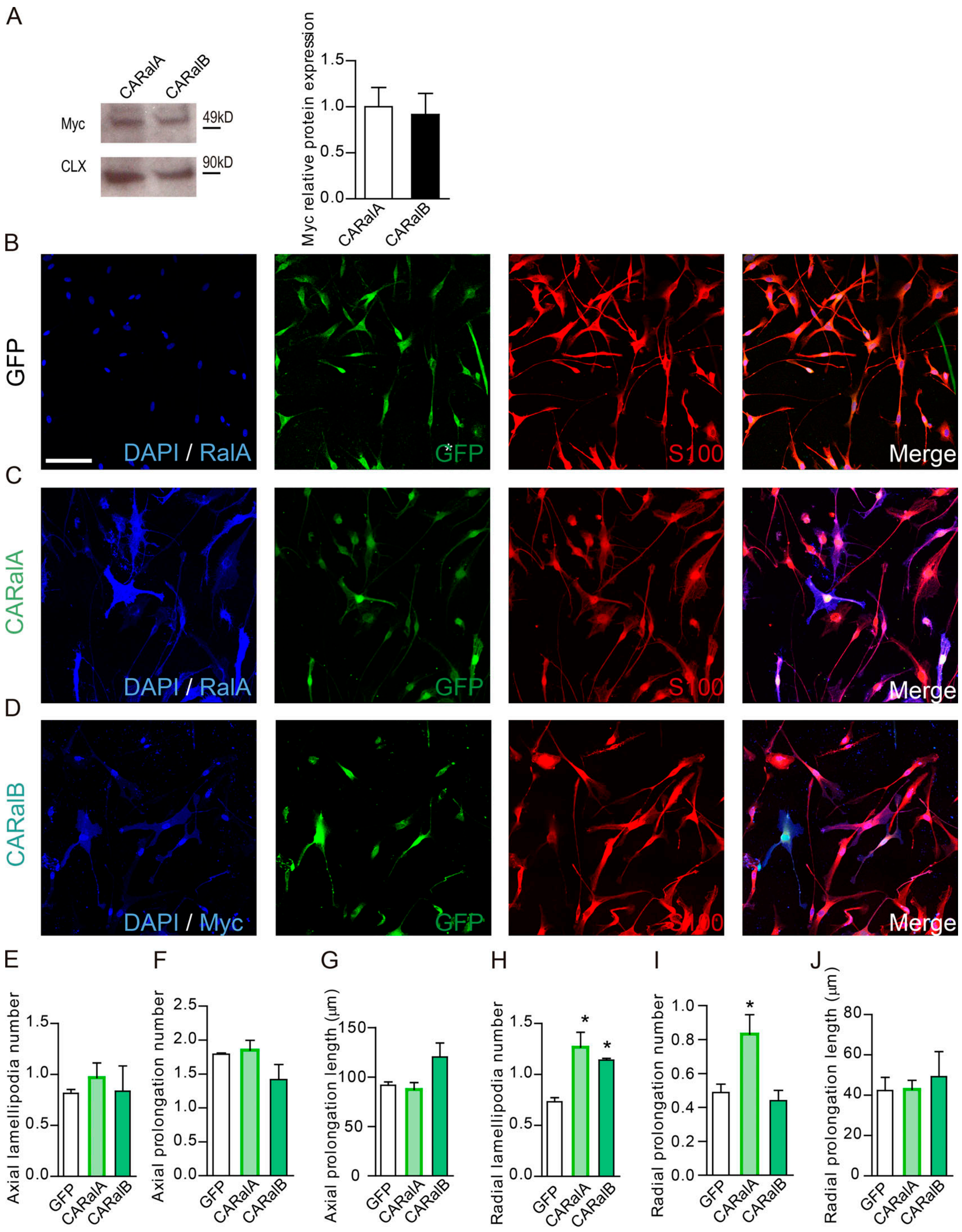

Figure S3. **Activated forms of RalGTPases increase radial SC process number without changes in axial process or in axial and radial process length. (A)** Western blot of the Myc epitope expression after transduction of SCs with CARalA and CARalB, respectively. Calnexin used as loading control. **(B–D)** Images of WT rat SC cultures transduced with lentivirus containing GFP as a control of transduction (B); CARalA, a constitutive form of RalA (C); and CARalB, a constitutive form of RalB (D). **(E–J)** Average of the number of axial (E) and radial (H) lamellipodia per cell, axial (F) and radial (I) prolongations, and axial (G) and radial (J) prolongation length quantification. GFP antibody is used as a marker of transduced cells labeled in green; S100 is used as a SC marker labeled in red. RalA antibody is used to demonstrate RalA-transduced cells and a Myc antibody to demonstrate RalB-transduced cells (both labeled in blue). DAPI is used as a nuclear marker. n = 3–6 pictures of three independent experiments for each virus condition. Data are presented as mean ± SEM (*, $P < 0.05$, one-way ANOVA, post hoc Tukey's used to compare with GFP). SC processes number and length are quantified in S100 channel for all S100-positive cells in GFP-transduced SCs and in RalA/Myc-positive cells in CARalA and CARalB. Scale bar, 50 µm.

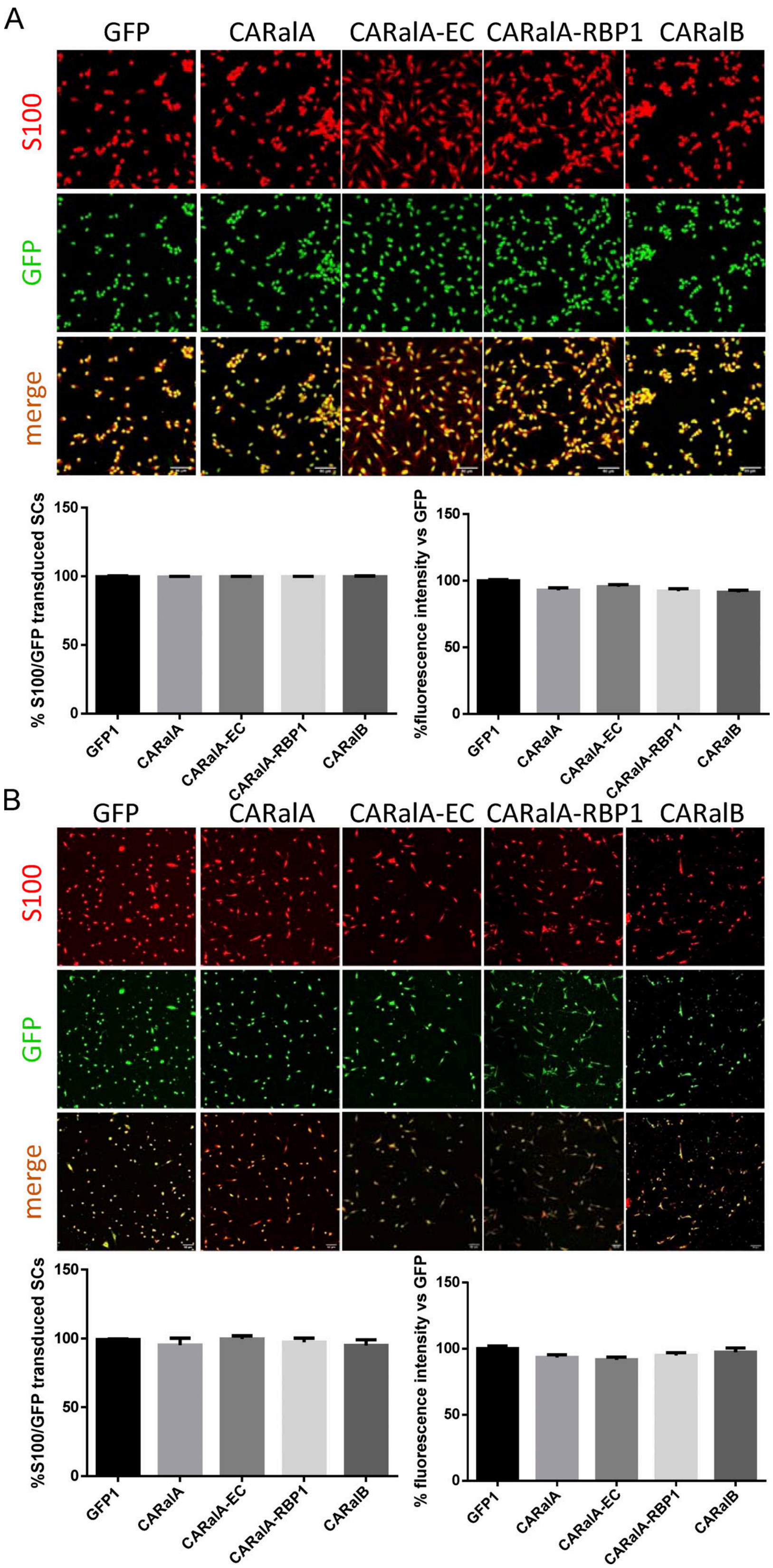

Figure S4. **Efficiency of transduction study. (A)** Pictures of rat SCs transduced with viral RalGTPases: GFP (control), CARalA, CARalA-EC, CARalA-RBP1, and CARalB. Efificency of transduction was reported as percentage of S100 cells/GFP-positive (n = 4) and also as percentage of fluorescence of GFP measured in single cells; n = 50 cells analyzed per group. Scale bar, 50 µm. **(B)** Pictures of mice SCs transduced with viral RalGTPases: GFP (control), CARalA, CARalA-EC, CARalA-RBP1, and CARalB. Efficiency of transduction was reported as a percentage of S100 cells/GFP-positive (n = 4) and also as percentage of fluorescence of GFP measured in single cells; n = 50 cells analyzed per group. Scale bars, 50 µm. Data are presented as mean ± SEM.

https://doi.org/10.1083/jcb.201811002

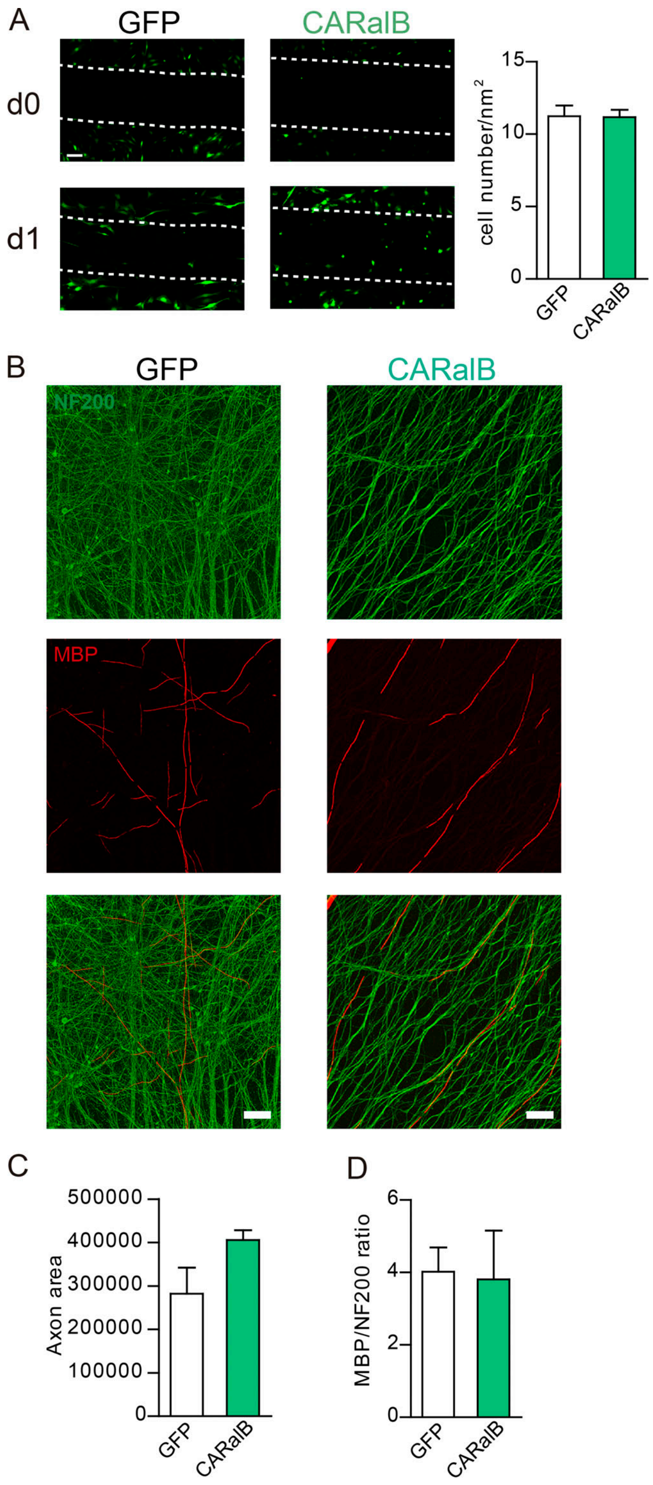

Figure S5. **RalB gain of function is not altering SC migration and myelination. (A)** Images of the wound healing assay of SCs transduced with lentivirus containing the activated form of RalB versus control followed by thequantification of the average of the number of cells invading the wound area. **(B)** Images of human iPSC-derived sensory neurons co-cultured with SCs transduced with lentivirus containing activated forms of RalB. Viral transduction had no effect on either axon outgrowth (C) or myelination of human iPSC-derived sensory neurons (D). Data are presented as mean ± SEM. Scale bars, 50 µm for migration pictures and 100 µm for co-cultures.
